# Acute gastroenteritis and the usage pattern of antibiotics and traditional herbal medications for its management in a Nigerian community

**DOI:** 10.1371/journal.pone.0257837

**Published:** 2021-10-04

**Authors:** Oluwapelumi Olufemi Adeyemi, Ade Stephen Alabi, Oluwasanmi Anuoluwapo Adeyemi, Olaoluwa Temitope Talabi, Oreoluwa M. Abidakun, Ireoluwa Yinka Joel, Nicola J. Stonehouse

**Affiliations:** 1 Department of Medical Microbiology and Parasitology, Faculty of Basic Clinical Sciences, College of Health Sciences, University of Ilorin, Ilorin, Nigeria; 2 School of Molecular and Cellular Biology and Astbury Centre for Structural Molecular Biology, Garstang Building, Faculty of Biological Sciences, University of Leeds, Leeds, United Kingdom; 3 Department of Anatomy, Faculty of Basic Medical Sciences, College of Health Sciences, University of Ilorin, Ilorin, Nigeria; 4 Department of Microbiology, Faculty of Life Sciences, Ajayi Crowther University, Oyo, Nigeria; 5 Department of Biochemistry, Faculty of Basic Medical Sciences, College of Medicine, University of Lagos, Idi-Araba, Lagos, Nigeria; 6 Central Research Laboratory, University of Ilorin, Ilorin, Nigeria; University of North Dakota, UNITED STATES

## Abstract

Acute gastroenteritis (AGE) is the highest cause of mortality worldwide in children under the age of 5 years, with the highest mortalities occurring in low-to-middle income countries. Treatment can involve use of unregulated herbal medication and antibiotics. A cross sectional study was carried out to investigate the use of antibiotics and traditional herbal medications in the management of AGE among Yòrùbá-speaking communities in Kwara State, Nigeria. Our findings suggest habitual use of antibiotics (54.6%) and herbal medication (42.5%) in the management of AGE with high levels of self-prescription of antibiotics (21.7%) and herbal medications (36.2%) within the community. Ethanolic extracts of selected herbal plants reported (i.e. *Aristolochia ringens*, *Azadirachta indica*, *Chromolaena odorata*, *Etanda Africana*, *Ficus capensis*, *Ficus vogelii*, *Mangifera indica*, *Momordica charantia*, *Ocimum gratisimum*, *Senna alata*, *Sorghum bicolor* and *Vernonia amygdalina*) were investigated for antibacterial properties, using bacteria known to be causative agents of AGE. Our findings showed that, with exception of *Ficus vogelii*, which enhanced bacterial growth, the plant extracts reported all showed some antibacterial activity. We further discuss our findings within a regulatory context, with the aim to guide the use of traditional and herbal medication in low-to medium income countries (LMICs) and reduce the potential risks associated with the development of antimicrobial resistance.

## Introduction

Acute gastroenteritis (AGE) is the highest cause of morbidity and mortality in children under the age of 5 in low-to-middle income countries (LMIC) and has significant impact on healthcare services in more developed countries [1]. AGE can be caused by infection with helminths, protozoa, bacteria and viruses with a seasonal pattern [2] of peak viral disease during the cooler seasons and bacterial disease in warmer months within tropical regions like Africa [2, 3]. Across all age groups, worldwide, noroviruses are responsible for most cases of viral gastroenteritis and remain the leading cause of AGE [4]. There is therefore no cure for many cases of AGE and clinical management relies on supportive therapy [5], which is often inaccessible in resource-limited LMICs [6].

The indigenous nature of African traditional medicines is founded on theories, beliefs and experiences but often overlaps with herbal medicine for diagnostic, prophylactic and therapeutic physical and mental healthcare [7]. Traditionally, African herbal medicines are prepared as alcohol infusions or tea brewed from part(s) of plants [8], with therapeutic benefits on infectious and non-infectious diseases derived from vast deposits of bioactive compounds with potent antimicrobial activities [9]. While some herbal medications are mixtures of various antimicrobial compounds, others are standalone herbs with complex antimicrobial properties. However, issues of toxicity continue to raise valid concerns owing to the handed-down tradition of self-prescription [10], erroneous use of incorrect plant species [11] and the lack of regulations on usage [12].

The gut microbiome is suggested to play a unique role in the outcome of gastrointestinal viral and bacterial infections through the maintenance of host physiological homeostasis, modulation of the host immune system, and interactions with pathogenic microbes [13, 14]. Therefore, the depletion of host microbiome through the overuse of broad-spectrum antibiotics [15] may result in gastrointestinal disease. In Sub-Saharan Africa, the unregulated use of herbal medicines together with antibiotic use [12] may contribute to the high mortalities of AGE and contribute to increased antimicrobial-resistance [16, 17]. Here, a descriptive cross-sectional survey was carried out to investigate local practices in the management of AGE in Kwara state, Nigeria, which include the use of antibiotics and traditional herbal medications. The study further investigated antibacterial properties of selected plants reported, to assess the potential impact of such practices.

## Materials and methods

### Ethical issues

Ethical approval was obtained from the Ethical Review Committee of the University of Ilorin Teaching Hospital with approval number ERC PAN/2019/09/1943. Respondents were informed of the aims, implications, and benefits of the study as well as their rights to withdraw at any point of the study. A written informed consent for voluntary participation in the study was obtained from each participant (or parents or guardians of underage participants) before enlistment in the study.

### Study area

Kwara State is located within the North-central geopolitical zone of the Federal Republic of Nigeria. It is sub-divided into 16 local government areas (LGAs) that comprise predominantly Yòrùbá [18] people that occupy 12 LGAs across the central and southern regions of Kwara; the Nupe, that occupy 2 LGAs in the eastern regions of Kwara; as well as Bariba and Fulani that occupy 2 LGAs in the Northern regions [19]. Kwara has a total landmass of 32,500 km^2^ and an estimated population size of 3.2 million people [20] that comprises Muslims, Christians, and believers and/or followers of indigenous African religions (i.e. African Traditional worshippers) [21].

### Target population

The study involved male and female outpatients aged 14 years and above on health visits to primary healthcare facilities in 4 randomly selected LGAs across the predominantly Yòrùbá central and southern regions. These regions were Ifelodun, Ilorin East, Isin and Oyun LGA, which together have an estimated adult population of 330,545 [20].

### Study design

A descriptive cross-sectional survey was carried out from September to November 2019. A sample size of 384 was determined using the Fisher’s formula [22] and a repartition rate of 12% added. The eligibility criteria included individuals that were aged 14 years and above that attended the study sites for cases of acute gastroenteritis. In total, 430 individuals were recruited by stratified non-probability convenience sampling [23] across the target LGAs. A set of pre-tested and standardised semi-structured questionnaire was administered to informed and consented respondents by trained research assistants during face-to-face personal interviews conducted in local languages understood by each respondent. Of the recruited respondents, 386 completed the survey.

### Plant harvest and extraction

Herbal plant leaves, stem or root prescriptions reported by the respondents for management of AGE were re-confirmed by traditional herbalists and locally sourced in Kwara, Nigeria (S1 Table and S1 Fig) between October and December 2020. Plants were taxonomically confirmed and identified at the Plant Biology Herbarium, University of Ilorin, Ilorin, Nigeria. The parts of plants that were prescribed were harvested, washed, dried at 16°C for 4 days and transferred to 45°C for 1 day prior to pulverisation. Pulverised plants were taken through a 3-step extraction process of cold maceration in absolute ethanol (200g/L) for 72 hours, filtration through Whatman filters and concentration at 45°C in a water bath. The 3-step extraction process was repeated three times to ensure exhaustive extraction. Total extraction yield was estimated as the % mass of concentrate recovered from the macerated input (i.e. % wet weight). Plant extracts were stored at 4°C until use.

### Bacterial culture

Laboratory reference strains of *Staphylococcus aureus* (*S*. *aureus*, ATCC-25923), Methicillin-resistant *Staphylococcus aureus* (MRSA, ATCC-43300) and *Escherichia coli* (*E*. *coli*, ATCC-29322) were sourced from the University of Ilorin Teaching Hospital, Ilorin, Nigeria; and laboratory isolates of *Pseudomonas aeruginosa* (*P*. *aeruginosa*), *Bacillus subtilis* (*B*. *subtilis*) and *Klebsiella pneumoniae* (*K*. *pneumoniae*) were sourced from Biological Sciences Faculty, Ajayi Crowther University, Oyo, Nigeria. Bacterial stocks were grown in nutrient broth (10 g/L Tryptone, 10 g/L NaCl and 5 g/L yeast extract) for 24 hours with shaking at 160 rpm and bacterial colonies were enumerated on nutrient agar using the pour plate method.

### Antibacterial assay

Duplicate wells of sterile flat bottom 96-well microtitre plates were seeded with 216.5 μl nutrient broth, 8.5 μl bacterial inoculum and 25 μl plant ethanolic (EtOH) extract to a final concentration of 100 mg/ ml in a total volume of 250 μl per well. Plates were covered with Parafilm and incubated at 37°C with shaking at 140 rpm for 18 hours. Bacterial growth was estimated as difference between the pre- and post-incubation turbidity using spectrophotometric (OD λ = 625 nm) measurements.

### Minimal Inhibitory Concentrations (MIC) and half maximal inhibitory concentration (IC_50_)

To determine the MIC of plant extracts, antibacterial assays were carried out within 96-well microtitre plates, using two-fold dilutions for each plant extract from 0.625mg/ml to 100 mg/ml. Plates were incubated at 37°C for 24 hours with shaking at 160 rpm. To estimate the IC_50_, values were fitted into a non-linear regression (asymmetric sigmoidal) dose-response curve using the GraphPad Prism version 7.01 for Windows (GraphPad Software, La Jolla CA).

### Data analysis

Normal continuous variables were described by means, binary variables were described by means of frequencies and percentages (%), while the relationships between categorical variables were tested by Pearson χ^2^ test at 95% confidence interval using the IBM SPSS Statistics 21 (Armonk, New York). A p-value of <0.05 was considered statistically significant. Two-tailed unpaired Student’s t-tests were carried out to measure difference between variables using GraphPad Prism version 7.01 for Windows (GraphPad Software, La Jolla CA). Significant differences between antibiotic and/or herbal-treated and non-treated respondent’s or plant extract-treated and non-treated bacterial strains are shown as p-values <0.05 (*), <0.01 (**) and <0.001 (***). Error bars shown represent standard error of the mean (S.E.M.) of respondents’ or multiple biological experiments, as stated.

## Results

### Socio-demographic profile of respondents

To investigate antibiotics and traditional herbal medications use in the management of AGE in Kwara State, Nigeria, a descriptive cross-sectional study was carried out in Ifelodun, Ilorin East, Isin and Oyun Local government areas, which are predominantly Yòrùbá communities within Kwara. The respondents identified as male (29.53%) and female (54.9%), while several respondents did not respond to this question (15.57%). The age of the respondents ranged from 14–88, (14–24 (25.91%), 25–40 (24.61%), 41–60 (17.1%) and 61–88 (10.62%); while a proportion (21.76%) of the respondents did not disclose their age. Their religious beliefs included Islam (52.33%), Christianity (36.27%), African Traditional Religion (2.33%) and the non-religious (9.07%).

### Treatment regimens for gastroenteritis

**Fig 1:**
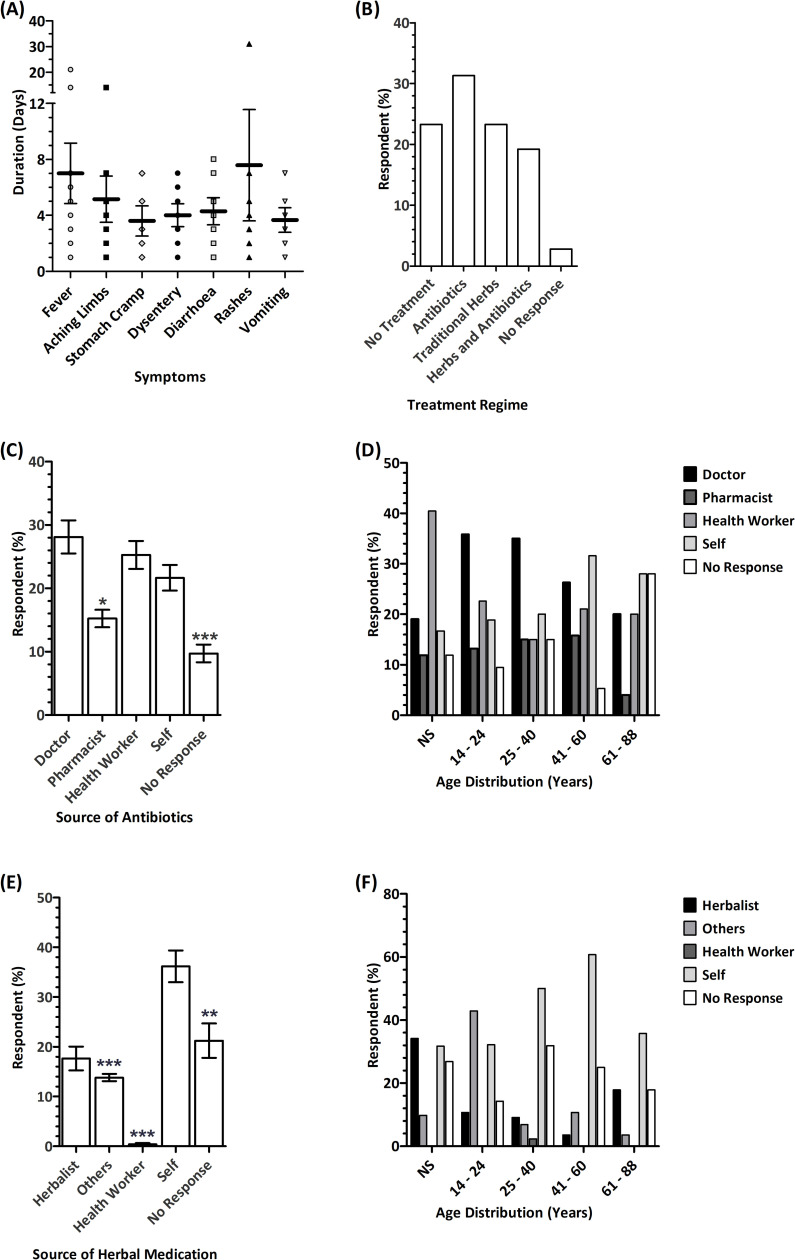
Reported symptoms, treatment regimens and sources of antibiotics and herbal medications. (A) Duration of reported symptoms of fever, aching limbs, stomach cramps, dysentery, diarrhoea, rash, and vomiting. (B) Treatment regime reported by respondents; (C) Source of antibiotic for treatment of symptoms reported. Error bars represent standard error of mean across source of antibiotics for symptoms reported among antibiotics users (n = 7 ± S.E.M., *p < 0.05, ***p < 0.001 in comparison to self-prescription). (D) Age group distribution of antibiotic users. (NS = not specified, *X*^2^ [df = 20, *n* = 386] = 25.906, *p* = 0.169). (E) Source of herbal prescription for treatment of symptoms reported. Error bars represent standard error of mean across source of herbal medications for symptoms reported among herbal medicine users (n = 7 ± S.E.M., ***p < 0.001 compared to self-prescription). (F) Age group distribution of traditional herbal medicine users. (NS = not specified, *X*^2^ (df = 20, *n* = 386) = 52.823, *p* = 0.000).

Symptoms of gastroenteritis such as diarrhoea (17%), dysentery (10.8%), vomiting (9.6%), fever (19.1%) and stomach cramps (18.1%) as well as non-specific symptoms such as joint aches (18.2%) were reported in over 90% of respondents that participated in this study, and these symptoms lasted a range of durations of 1–31 days (Fig 1A). To investigate the management of AGE within the community, we assessed the treatment regimens reported by the respondents (Fig 1B and Table 1). Overall, a greater proportion of respondents (54.6%) took antibiotics, while 42.5% took herbal medication (Fig 1B). Furthermore, approximately 19% of the respondents combined the use of antibiotics and herbal medication and 23% used no medication.

**Table 1:** The source of antibiotics and traditional herbal medicine reported by respondents, for the symptoms listed.

**Symptoms**	**No antibiotic (%)** ^**a**^	**Source of antibiotics for treatment of AGE symptoms**							**Chi square (χ** ^**2**^ **)**		
		**Doctor (%)** ^**a**^	**Pharmacist (%)** ^**a**^	**Health Worker (%)** ^**a**^	**Self (%)** ^**a**^	**No Response (%)** ^**a**^	**Sub-total (%)** ^**a**^	**Total (%)** ^**a**^	**Value**	**df**	**p-value**
Stomach cramps	79 (41.6)	27 (14.2)	15 (7.9)	32 (16.8)	27 (14.2)	10 (5.3)	111 (53.2)	190 (100)	15.850	10	>0.05
Aching limbs	92 (47.7)	24 (12.4)	20 (10.4)	17 (8.8)	24 (12.4)	16 (8.3)	101 (44.0)	193 (100)	53.869	10	<0.0001
Rashes	40 (50.6)	12 (15.2)	7 (8.9)	12 (15.2)	6 (7.6)	2 (2.5)	39 (46.8)	79 (100)	50.098	10	<0.0001
Fever	93 (45.6)	34 (16.7)	18 (8.8)	24 (11.8)	22 (10.8)	13 (6.4)	111 (48.0)	204 (100)	55.282	10	<0.0001
Vomiting	45 (43.7)	24 (23.3)	5 (4.9)	13 (12.6)	9 (8.7)	7 (6.8)	58 (49.5)	103 (100)	10.956	10	>0.05
Diarrhoea	70 (39.3)	27 (15.2)	15 (8.4)	25 (14.0)	33 (18.5)	8 (4.5)	108 (56.2)	178 (100)	29.788	10	<0.001
Dysentery	42 (36.8)	15 (13.2)	12 (10.5)	24 (21.1)	16 (14.0)	5 (4.4)	72 (58.8)	114 (100)	27.612	10	<0.05
**Symptoms**	**No Herbs (%)** ^**a**^	**Source of herbs for treatment of AGE symptoms**							**Chi square (χ** ^**2**^ **)**		
		**Herbalist (%)** ^**a**^	**Other (%)** ^**a**^	**Health Worker (%)** ^**a**^	**Self (%)** ^**a**^	**No Response (%)** ^**a**^	**Sub-total (%)** ^**a**^	**Total (%)** ^**a**^	**Value**	**df**	**p-value**
Stomach cramps	94 (49.5)	24 (12.6)	0 (0.0)	15 (7.9)	37 (19.5)	20 (10.5)	96 (40.0)	190 (100)	28.598	10	<0.001
Aching limbs	99 (51.3)	12 (6.2)	0 (0.0)	13 (6.7)	41 (21.2)	28 (14.5)	94 (34.2)	193 (100)	100.881	10	<0.0001
Rashes	32 (40.5)	8 (10.1)	0 (0.0)	5 (6.3)	19 (24.1)	15 (19.0)	47 (40.5)	79 (100)	92.814	10	<0.0001
Fever	112 (54.9)	13 (6.4)	0 (0.0)	17 (8.3)	44 (21.6)	18 (8.8)	92 (36.3)	204 (100)	94.468	10	<0.0001
Vomiting	62 (60.2)	11 (10.7)	1 (1.0)	10 (9.7)	13 (12.6)	6 (5.8)	41 (34.0)	103 (100)	21.357	10	<0.05
Diarrhoea	92 (51.7)	12 (6.7)	1 (0.6)	12 (6.7)	41 (23.0)	20 (11.2)	86 (37.1)	178 (100)	9.955	10	>0.05
Dysentery	48 (42.1)	20 (17.5)	0 (0.0)	10 (8.8)	22 (19.3)	14 (12.3)	66 (45.6)	114 (100)	41.649	10	<0.0001

^a^ Percentage (%) within row (i.e. % among the sources of prescription across the various symptoms)

Of the respondents who used antibiotics in the treatment of the symptoms of gastroenteritis reported, self-prescription of antibiotics (21.7%) was significantly higher than prescriptions given by pharmacists (15.2%) or undisclosed sources (9.7%) (Fig 1C). Our data further showed that, as the age of respondents increased, there was a decrease in reliance on prescription of antibiotics by a doctor. Consistent with this, of the respondents who used herbal medicines in the treatment of the reported symptoms of gastroenteritis, self-prescription tended to increase with age but this was not statistically significant (Fig 1D). Formulation of traditional and herbal medicine is often undertaken by herbalists and the use of such remedies often relies on self-prescription. As expected, our data here showed that self-prescribed herbal medications (36.2%) were significantly higher than prescriptions given by herbalists (17.7%), family and acquaintances (i.e. others; 13.8%), healthcare workers (~1%), and undisclosed sources (i.e. no response, 21.2%; Fig 1E and Table 1). Furthermore, our data also showed that as the age of respondents increased, there was an increase in self-prescription and decrease in dependence on family and friends (i.e. others) for prescription among respondents (Fig 1F).

### Respondents’ approach to management of AGE

In this study, we focused on herbal and antibiotic treatment regimens. In some cases, this involved successive courses of antibiotics. We observed that of the users of antibiotics, metronidazole was more common across all courses of prescriptions (Table 2). Furthermore, we observed that some respondents used up to three courses of antibiotics (Table 2). Metronidazole (52%) and tetracycline (75%) were mainly self-prescribed, while penicillin derivatives such as amoxicillin (60%), ampicillin (57%) and Amplicox (i.e., a combination of ampicillin and cloxacillin) (57%) were prescribed mostly by a combination of doctors and healthcare workers. The only prescriptions of ceftriaxone, ciprofloxacin and chloramphenicol reported were by healthcare professionals, while all users of doxycycline and Phthalysulphathiazole (brand named Thalazole) were self-prescribed. Together, approximately 88% of respondents had no record of which antibiotics were prescribed by the health personnel, while 3% respondents did not know which antibiotics, they had self-prescribed (Table 2).

**Table 2:** Antibiotic courses reported by respondents.

Antibiotic	First antibiotic course				Second antibiotic course				Third antibiotic course			
	HP (%)^a^	Self (%)^a^	NR (%)^a^	Sub-Total (%)^b^	HP (%)^a^	Self (%)^a^	NR (%)^a^	Sub-Total (%)^b^	HP (%)^a^	Self (%)^a^	NR (%)^a^	Sub-Total (%)^b^
Metronidazole	27 (47)	26 (45)	5 (9)	58 (44)	4 (67)	2 (33)	0 (0)	6 (9)	1 (33)	2 (67)	0 (0)	3 (6)
Tetracycline	1 (25)	3 (75)	0 (0)	4 (3)	2 (25)	6 (75)	0 (0)	8 (12)	0 (0)	0 (0)	0 (0)	0 (0)
Ceftriaxone	1 (100)	0 (0)	0 (0)	1 (1)	0 (0)	0 (0)	0 (0)	0 (0)	0 (0)	0 (0)	0 (0)	0 (0)
Doxycycline	0 (0)	0 (0)	0 (0)	0 (0)	0 (0)	1 (100)	0 (0)	1 (1)	0 (0)	1 (100)	0 (0)	1 (2)
Amoxicillin	6 (86)	1 (14)	0 (0)	7 (5)	0 (0)	3 (100)	0 (0)	3 (4)	0 (0)	0 (0)	0 (0)	0 (0)
Ampicillin	2 (67)	1 (33)	0 (0)	3 (2)	1 (50)	1 (50)	0 (0)	2 (3)	1 (50)	1 (50)	0 (0)	2 (4)
Ciprofloxacin	7 (100)	0 (0)	0 (0)	7 (5)	1 (100)	0 (0)	0 (0)	1 (1)	0 (0)	0 (0)	0 (0)	0 (0)
Chloramphenicol	1 (100)	0 (0)	0 (0)	1 (1)	0 (0)	0 (0)	0 (0)	0 (0)	0 (0)	0 (0)	0 (0)	0 (0)
Phthalysulphathiazole	0 (0)	1 (100)	0 (0)	1 (1)	0 (0)	0 (0)	0 (0)	0 (0)	0 (0)	0 (0)	0 (0)	0 (0)
Combination of Ampicillin and Cloxacillin (Ampliclox)	2 (40)	3 (60)	0 (0)	5 (4)	2 (100)	0 (0)	0 (0)	2 (3)	0 (0)	0 (0)	0 (0)	0 (0)
Unknown	41 (89)	1 (2)	4 (9)	46 (35)	39 (87)	2 (4)	4 (9)	45 (66)	38 (88)	1 (2)	4 (9)	43 (88)
**Overall**	**Total**			**133 (100)**	**Total**			**68 (100)**	**Total**			**49 (100)**

HP: Healthcare personnel (i.e. combined prescriptions from doctors, pharmacists and health workers); NR: No response.

^a^ Percentage (%) within row (i.e. % among the sources of prescription across the various symptoms), ^b^ percentage across column. First treatment course: χ^2^ (df = 55, *n* = 386) = 488.97, *p* < 0.0001; Second treatment course: χ^2^ (df = 45, *n* = 386) = 261.46, *p* < 0.0001; Third treatment course: χ^2^ (df = 30, *n* = 386) = 191.67, *p* < 0.0001

Self-prescription of herbal mixtures has a long history in Africa. Hence, we sought to investigate the usage of the herbal medications here. From our findings, some herbs were mainly used as single course medications such as Agbo iba, ’Agbo jedi-jedi’, Dutchman’s pipe, Akintola leaf (*Chromolaena odorata*), Mango leaf (*Mangifera indica*), Drumstick tree leaves and Bitter leaf (*Vernonia amygdalina*). However, Dongoyaro leaves (*Azadirachta indica*) and Goko Herbal Cleanser were used in combinations with other herbs as second course herbal medications. Additionally, Roscoe was mainly used as the third of three herbal medication options (Table 3).

**Table 3:** Traditional herbal regimens taken by respondents.

SN	Herb/ herbal mixture	Latin Name	Treatment Course		
			No. of respondents using herbs as first course (%)	No. of respondents using herbs as second course (%)	No. of respondents using herbs as third course (%)
1.	Agbo iba [24]:	N/A	40 (93.0)	3 (7.0)	0 (0)
	Pineapple bark	*Ananas comosus*			
	Pawpaw (seed and leaf)	*Carica papaya*			
	Neem tree (Dongoyaro)	*Azadirachta indica*			
	Lime juice	*Citrus aurantifolia*			
	Lemon grass	*Cymbopogon citrates*			
	Guava leaves	*Psidium guajava*			
	Scented leaves (Efirin)	*Ocimum gratisimum*			
2.	Marike (Spindle tree)	*Euonymus europaeus*	1 (100.0)	0 (0)	0 (0)
3.	Cacia	*Cassia alata*	1 (16.7)	1 (16.7)	4 (66.7)
4.	Alagau	*Gynura procumbens*	0 (0)	1 (50.0)	1 (50,0)
5.	Dutchman’s pipe (Akogun)	*Aristolochia ringens*	4 (57.1)	1 (14.3)	2 (28.6)
6.	Periplocaceae leaf (Ogbo)	*Parquetina nigresceus*	4 (50.0)	4 (50.0)	0 (0)
7.	Igi odan (leaves)	*Ficus capensis*	1 (50.0)	1 (50.0)	0 (0)
		*Ficus vogelii* synonym *F*. *lutea*			
8.	Akintola (leaves)	*Chromolaena odorata*	1 (100.0)	0 (0)	0 (0)
9.	Dongoyaro (Neem tree) leaves	*Azadirachta indica*	2 (40.0)	3 (60.0)	0 (0)
10.	’Agbo jedi-jedi’ [24]:	N/A	23 (82.1)	4 (14.3)	1 (3.6)
	Scented leaves (efirin)	*Ocimum gratisimum*			
	Grapefruit leaves	*Citrus paradise*			
	Bitter leaf (Ewuro)	*Vernonia amygdalina*			
	Sorghum leaves	*Sorghum bicolor*			
	Garlic	*Allium sativum*			
	Naphthalene tablets	N/A			
11.	Ewe Ajarere	*Senna alata*	1 (50.0)	1 (50.0)	0 (0)
12.	Awopa (leaf and bark)	*Enantia chlorantha*	0 (0)	4 (100.0)	0 (0)
13.	Igbaluwere	*Etanda africana*	7 (58.3)	2 (16.7)	3 (25.0)
14.	Mango leaf	*Mangifera indica*	7 (87.5)	1 (12.5)	0 (0)
15.	Gangaria de flush [25]	N/A	2 (100)	0 (0)	0 (0)
	Lablab	*Dolichos lablab*			
	African Mahogany (bark)	*Khaya grandifolia*			
	Fresen (leaf)	*Securidaca longepedunculata*			
	Cucurbitaceae (leaf)	*Citrullus colocynthis*			
	African Star Apple (seeds)	*Chrysophyllum albidum*			
	Schum and Thonn	*Curciligo pilosa*			
	Sorghum leaves and seed	*Sorghum bicolor*			
16.	Moringa (Drumstick) tree.	*Moringa oleifera*	1 (100)	0 (0)	0 (0)
17.	Filasco	*Sena alata*	5 (100)	0 (0)	0 (0)
18.	Roscoe (Atare)	*Aframomum melegueta*	0 (0)	0 (0)	1 (100.0)
19.	Goko Herbal Cleanser	N/A	2 (2.8)	70 (97.2)	0 (0)
	Bitter leaf (Ewuro)	*Vernonia amygdalina*			
	Pigeon pea (Otili) leaves	*Cajanus cajan*			
	Ginger	*Zingiber officinale*			
	Garlic	*Allium sativum*			
	Cane sugar	*Saccharum officinarum*			
	Brown sugar syrup	Caramel			
20.	Bitter leaf (Ewuro)	*Vernonia amygdalina*	11 (68.8)	5 (31.3)	0 (0)
21.	Kakan furu	*Sizygium aromaticum*	1 (50.0)	1 (50.0)	0 (0)
22.	None	N/A	202	284	303
23.	No response	N/A	70	70	71
24.	**Total**		**386**	**386**	**386**

Percentage (%) values represent values represent proportions within the sum of all three courses of respective antibiotic use. First treatment course: χ^2^ (df = 100, n = 386) = 532.56, p < 0.0001; second treatment course: χ^2^ (df = 75, n = 386) = 198.13 p < 0.0001; third treatment course: χ^2^ (df = 35, n = 386) = 110.79, p < 0.0001. N/A (not applicable).

### Antibacterial efficacies of herbal plants

**Fig 2:**
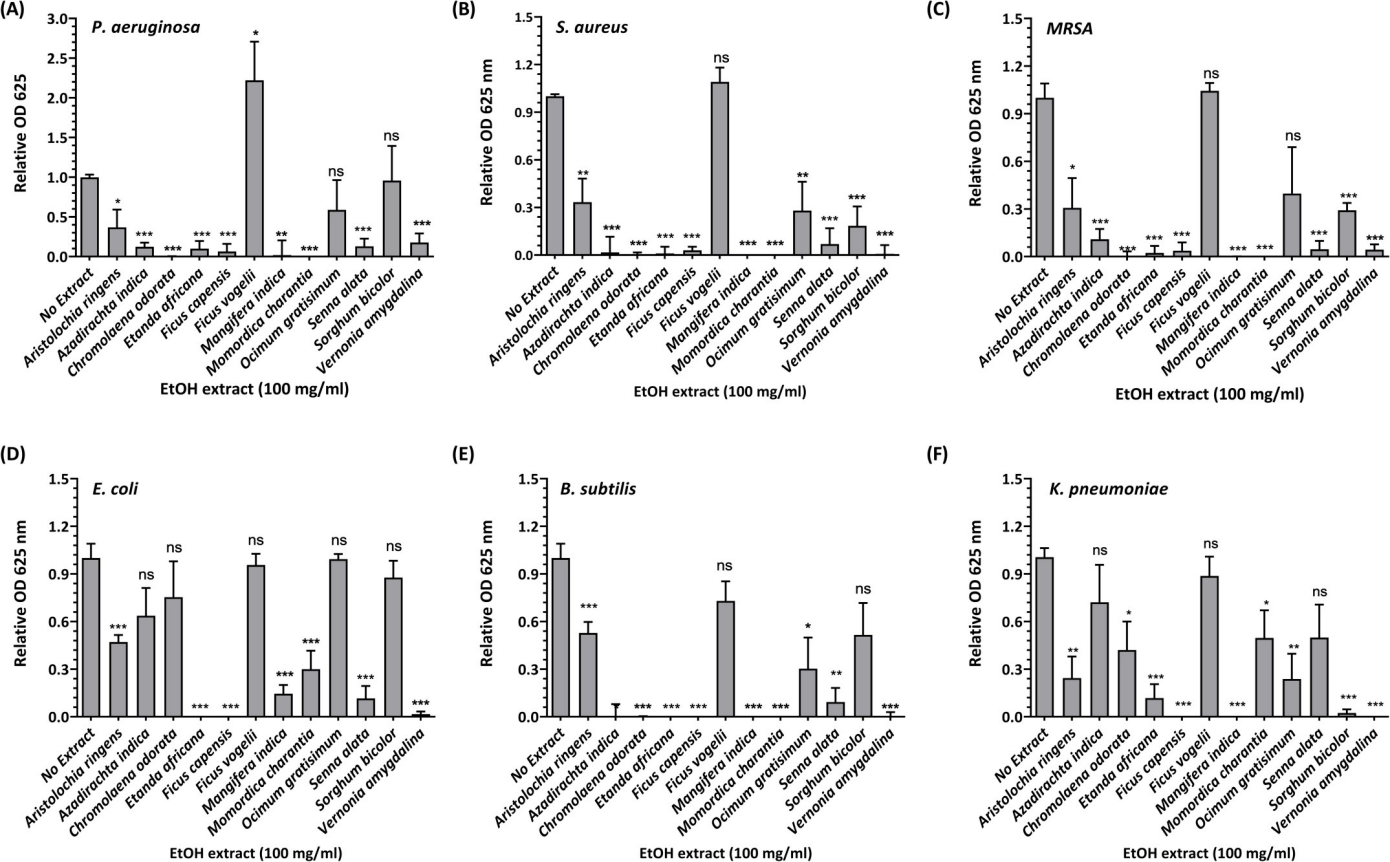
Bacterial inhibition by plant ethanolic extracts. Duplicate wells of 96 well microplates were seeded to a final volume of 250 μl comprising 25 μl plant ethanolic (EtOH) extract, 8.5 μl bacteria and 216.5 μl nutrient broth. Plates were incubated at 37°C with shaking at 140 rpm for 18 hours. Bacterial growth is shown as spectrophotometric (OD λ = 625 nm) measurement of media turbidity measured against pre-incubation reads. Each panel shows the relative OD of treated samples compared to non-treated samples of (A) *P*. *aeruginosa*, (B) *S*. *aureus* (C) MRSA (D) *E*. *coli* (E) *B*. *subtilis*, and (F) *K*. *pneumoniae* (n = 2 ± S.E.M., ns = not significant, *p<0.05, **p<0.01, ***P<0.001).

Some herbal medications reported in this study such as Agbo iba, ’Agbo jedi-jedi’, Gangaria de flush and Goko herbal cleanser are mixtures of multiple herbs, while others are individual herbs (Table 3), some of which have been reported for antidiarrheal properties using castor oil-induced diarrhoea in Wistar rats [26–34]. In this report, we sought to investigate the impact of the unregulated use of herbal plants on AGE using *in vitro* assays to assess the antibacterial effects of ethanolic extracts of some of the reported plants. These were sourced from local traditional herbalists and taxonomically identified (S1 Table and S1 Fig). Gram positive (*S*. *aureus*, MRSA and *B*. *subtilis*) and Gram negative (*P*. *aeruginosa*, *E*. *coli* and *K*. *pneumoniae*) bacteria were seeded into duplicate wells of 96-well microplates at a concentration of 40.7 (± 27.3 S.D.) colony forming units per millilitre (CFU/ml) in the presence and absence of 100 mg/ml plant extracts. Bacterial growth or inhibition was measured as broth turbidity at OD λ = 625 nm (Fig 2). Our data showed that the ethanolic leaf extracts of *Etanda africana*, *Ficus capensis*, *Mangifera indica* and *Vernonia amygdalina* inhibited the growth of all bacterial species tested. *Aristolochia ringens* showed moderate inhibitory potentials across bacterial species tested except for *P*. *aeruginosa* which was strongly inhibited by *A*. *ringens*. *Senna alata* showed strong inhibitory properties across bacterial species tested except for *K*. *pneumoniae*. *Azadirachta indica*, *Chromolaena odorata* and *Momordica charantia* more efficiently inhibited the growth of Gram-positive bacteria. Our data further showed that *Ocimum gratisimum*, *Sorghum bicolor* and *Terminalia avicennioides* showed weak inhibitory properties across bacterial species tested. *F*. *vogelii* was non-inhibitory and significantly (p<0.05) enhanced the growth of *P*. *aeruginosa*.

We further assessed the minimal and half-maximal inhibitory concentrations (MIC and IC_50_) using a range of concentrations from 0.625 mg/ml to 100 mg/ml. Our data showed that *E*. *Africana*, *F*. *capensis*, *M*. *indica*, *S*. *alata* and *V*. *amygdalina* resulted in low MICs and IC_50_ values across all bacterial species tested; *M*. *charantia* resulted in low MIC and IC_50_ values across all bacteria except *E*. *coli*, while other plants extracts showed moderate to high MICs and IC_50_ across bacterial species tested (Table 4).

**Table 4:** Antimicrobial activity of selected plants extracts.

Plant extract (mg/ml)	*Test bacteria*											
	*P*. *aeruginosa*		*S*. *aureus*		*MRSA*		*E*. *coli*		*B*. *subtilis*		*K*. *pneumoniae*	
	MIC	IC_50_	MIC	IC_50_	MIC	IC_50_	MIC	IC_50_	MIC	IC_50_	MIC	IC_50_
*Aristolochia ringens*	100.0	--	100.0	43.31	50.0	12.55	100.0	25.58	100.0	--	100.0	12.85
*Azadirachta indica*	100.0	--	25.0	17.88	25.0	21.81	--	--	12.5	18.56	NA	50.21
*Chromolaena odorata*	50.0	25.55	50.0	26.34	12.50	17.71	--	--	--	--	50.0	30.17
*Etanda Africana*	1.25	2.65	2.5	1.35	1.25	0.31	1.25	0.41	1.25	0.23	0.625	0.18
*Ficus capensis*	2.50	2.65	2.5	2.41	5.0	4.88	2.50	1.51	2.50	0.68	5.0	4.81
*Ficus vogelii*	--	--	--	--	--	--	--	--	--	--	--	--
*Mangifera indica*	6.25	7.22	10.0	9.55	6.25	3.0	6.25	3.66	12.5	7.22	5.0	2.14
*Momordica charantia*	6.25	0.90	6.25	5.46	6.25	5.29	100.0	65.39	6.25	0.90	6.25	4.51
*Ocimum gratisimum*	--	--	100.0	67.19	--	--	--	--	--	--	100	--
*Senna alata*	5.0	2.65	5.0	4.49	2.50	2.14	12.5	5.21	10.0	1.17	5.0	12.17
*Sorghum bicolor*	--	--	100.0	45.92	25.0	17.72	--	--	--	--	100.0	54.89
*Vernonia amygdalina*	12.05	6.53	6.25	2.84	6.25	5.84	12.5	11.19	12.5	6.53	12.5	12.20

MIC (minimal inhibitory concentration), IC_50_ (half maximal inhibitory concentration)

## Discussion

The seasonal pattern of AGE [2] suggest peak periods of viral gastroenteritis during the cooler rainy months of March to October in Nigeria [35] with higher occurrences in July and October across Africa [2] and peak bacterial gastroenteritis in the warmer months from November to March [3]. This study was undertaken in primary healthcare facilities during a period when viral gastroenteritis would predominate. It highlights a high level of antibiotic and herbal medicine use during the study period that are likely to have little benefit to patients with viral disease.

Although the use of antibiotics could be efficient in about 20% cases of bacterial AGE they are of no documented benefit in the management of protozoans, helminths, or viruses (which account for 70% of AGE in children) [36]. As a LMIC, Nigeria is reported to have a high level of drugs prescription per encounter [37], most of which occur via primary healthcare settings [38]. Antibiotics are classified bacteriostatic or bactericidal, while antimicrobials could be antibacterial, fungicidal, antiviral, antiprotozoal, etc [39]. This study reported the use of Metronidazole as most common across all courses of prescriptions (Table 2). As a narrow spectrum nucleic acid inhibitor, Metronidazole is active against enteropathogenic bacteria and protozoans [40] with growing concerns about Metronidazole-resistant organisms [41, 42]. The overuse of antibiotics in humans (as well as for animals) has led to the emergence of multidrug-resistant bacteria [43]. However, it seems clear that overuse of other antimicrobials could also be of concern. Overall, this study found that 3 in 4 cases of AGE were managed with the use of antimicrobials with 1 in 2 cases managed with the use of antibiotics. Overall, 1 in 5 prescriptions of antibiotics and 1 in 3 prescriptions of antimicrobial herbal medications were self-generated by respondents, as expected with indigenous traditional practices [10]. This study also showed that most visits to the primary health centres within the study areas were secondary, possibly following ineffective self-prescriptions of antimicrobials. These figures obtained from primary healthcare facilities could be extrapolated to indicate high levels of antimicrobial use within the larger society, and in particular, the common usage of antibiotics.

In Africa, the use of traditional herbal medicines or indigenous healthcare, cut across religious beliefs. While some traditional and herbal remedies are curative, there have been increasing cases of adverse reactions and contraindicative interactions with orthodox drugs [44, 45] due to poorly understood pharmacological effects [11]. Although some herbal mixtures used by respondents in this study are known antimicrobials, others include hepatotoxic [46] as well as other toxic [47] components (Table 3). This study suggests the need for regulatory guidelines on the use of antimicrobial herbs.

The primary healthcare facilities investigated in this study did not carry out laboratory diagnosis of reported cases of AGE or follow up the patients. Previous studies, using castor oil-induced diarrhoea in Wistar rats, have reported antidiarrheal properties of a number of herbal extracts [26, 30, 32, 48–50]. Therefore, we chose to investigate the antibacterial properties of selected plants, which were major ingredients of herbal medication reported as used in the management of AGE. Antibacterial properties of the selected plant extracts were investigated using samples of bacteria known to be causative agents of gastroenteritis.

Leaf extracts of Neem tree (*A*. *indica*) constitute a vital component of the popular concoction, Agbo-iba, which is commonly used as a febrile prophylactic especially in the treatment of malaria [24], and recently reported to have antiviral effects on type-2 Dengue virus [51] and group B coxsackie viruses known to cause gastroenteritis [52]. However, few respondents reported the use of *A*. *indica* alone for the management of AGE. Our bacterial assays showed that *A*. *indica* did not inhibit the growth of coliforms (*E*. *coli* and *K*. *pneumoniae*) but showed strong inhibitory effects on other bacterial samples investigated (Fig 2). Although *C*. *odorata* was not popular among respondents, during our herbal harvest (Table 3), it was prescribed as an AGE prophylactic by a local herbalist and therefore investigated. Like *A*. *indica*, leaf extracts of *C*. *odorata* as well as *M*. *charantia* showed limited inhibitory effects on coliforms but strong inhibitory effects on other bacteria. This suggests its usefulness as a possible antibacterial against non-coliforms. Root extracts of *A*. *ringens* was not a popular herbal option among respondents (Table 3), however, locally used in the management of a wide range of unrelated conditions that cut across inflammatory and noninflammatory anomalies as well as infectious and non-infectious diseases [53]. The assays, however, showed that this had moderate antibacterial activities across all bacterial samples tested here (Fig 2). In agreement with this, the antidiarrheal properties of *A*. *indica* [48], *C*. *odorata* [26], *M*. *charantia* [32] and *A*. *ringens* [49] have been reported elsewhere.

Leaf extracts of *O*. *gratisimum* as well as sheath extracts of *S*. *bicolor* showed non-inhibitory effects against two-third of the Gram-negative bacterial agents of AGE investigated. While *O*. *gratisimum* moderately inhibited the growth of *S*. *aureus* (but not the antibiotic-resistant strain, *MRSA*), *B*. *subtilis* and *K*. *pneumoniae*; extracts of *S*. *bicolor* strongly inhibited the growth of *S*. *aureus*, moderately inhibited the growth of its antibiotic-resistant strain, *MRSA*, but strongly inhibited the growth of *K*. *pneumoniae* (Fig 2). This suggests some inhibitory similarities between both herbal extracts. Although, *S*. *bicolor* has been reported to have anti-inflammatory and immunomodulatory properties [51], antiviral effects of *O*. *gratisimum* against (the non-enteric) human immunodeficiency virus-1 (HIV-1) have been reported [54]. *O*. *gratisimum* is a common plant ingredient of two commonly used herbal mixtures, ‘Agbo iba’ and ’Agbo jedi-jedi’ [24]. Both were very popular among the respondents, however, ‘Agbo iba’ includes *A*. *indica* while ’Agbo jedi-jedi’ includes *V*. *amygdalina*, which both inhibited the growth of all bacterial samples investigated in this study (Fig 2). In addition to extracts of *O*. *gratisimum*, ’Agbo jedi-jedi’ [24] also includes *S*. *bicolour* and the more potent antibacterial, *V*. *amygdalina* (discussed below); thus suggesting its potential effectiveness against bacterial gastroenteritis. Although studies have reported on the antidiarrheal activities of *O*. *gratisimum* [30] and *V*. *amygdalina* leaf extracts [30] as well as seed extracts of *S*. *bicolor* [50], sheath extracts of *S*. *bicolor* have not been reported to have antidiarrheal activities.

Our findings further showed that extracts of *E*. *Africana*, Igi Odan (*F*. *capensis*), *M*. *indica* and *V*. *amygdalina* were strong inhibitors of all bacterial samples investigated. Studies have reported on antidiarrheal activities of *F*. *capensis* [55], *M*. *indica* [28] and *V*. *amygdalina* [27], and the antiviral effects of *M*. *indica* against non-enteric viruses such as herpes simplex virus (HSV)-1 and -2 and HIV (reviewed in [51]). Although extracts of *E*. *Africana*, *F*. *capensis* and *M*. *indica* are used as individual herbal medications, extracts of *V*. *amygdalina* was popularly used individually or as a constituent of the ’Agbo jedi-jedi’ concoction [24] (Table 3). However, among these antibacterial herbal extracts, Igi Odan, was reported by only one respondent. It is interesting to note that Igi Odan is a generic Yòrùbá name for tropical fig trees (*Terminalia* sp and *Ficus* sp [56]), which are mostly used as herbal treatments for infectious and non-infectious diseases. In this study, two locally common fig tree species (i.e. *F*. *vogelii* and *F*. *capensis*) were independently supplied by local herbalists as Igi Odan. Our investigations showed that while *F*. *capensis* had strong antibacterial properties across all bacterial agents investigated in this study, *F*. *vogelii* had no antibacterial properties, but significantly enhanced the growth of *P*. *aeruginosa* (Fig 2). We speculate that variation in the composition of Igi Odan may suggest why it was unpopular among respondents. Although it should be noted that our study did not include all potential causative agents of AGE, it highlights the need for regulations in the use of herbal remedies to prevent the potentially erroneous use of plant species [11].

Our findings (Table 4) also showed that some plants extracts investigated in this study (i.e. *E*. *Africana*, *F*. *capensis*, *M*. *indica*, *S*. *alata* and *V*. *amygdalina*) inhibited bacterial growth at relatively low concentrations (<6.25 mg/ml), irrespective of bacterial species, while others did not. This may be due to abundance of active compounds, which is likely to vary between individual plants of the same species. It should also be noted that the dosage of traditional herbal medications prescribed by herbalists is also likely to vary. Furthermore, investigation of any. Although investigating the antiviral properties of the plants extracts, will be informative to include in future studies, alongside using diagnostic tools and patient follow-up questionnaires, they were outside the remit of this study. However, these are challenging to undertake in a rural LMIC community.

## Conclusion

This study reports the use of antibiotics and herbs/herbal mixtures in the management of AGE within an African community, much of which is unregulated. Furthermore, it reveals inadequacies in the laboratory diagnosis and poor management of AGE in primary healthcare facilities. The findings from this study emphasise the need for further research on traditional and herbal medication, including investigation of anti-viral effects on viral agents of gastroenteritis. Monitoring the use of antimicrobials in LMICs and promoting public awareness would also be advantageous as over-use of antibiotics together with unregulated use of other antimicrobials has the potential to aggravate non-bacterial gastroenteritis and may explain the increasing burden of AGE in LMICs.

## Supporting information

S1 Table Examples of the plants used in the study. (DOCX)

S1 Fig Image of harvested plant parts. Figure shows (A) Image of *Aristolochia ringens* root (B) Upper and lower images of *Azadirachta indica* leaf (C) Upper and lower images of *Chromolaena odorata* leaf (D) Upper and lower images of *Etanda africana* leaf (E) Upper and lower of *Ficus capensis* leaf (F) Upper and lower images of *Ficus vogelii* (syn. F. lutea) leaf (G) Upper and lower images of *Mangifera indica* leaf (H) Upper and lower images of *Momordica charantia* leaf (I) Upper and lower images of *Ocimum gratisimum* leaf (J) Upper and lower images of *Senna alata* (K) Images of *Sorghum bicolor* sheath (L) Upper and lower images of *Vernonia amygdalina* leaf.
(TIF)

S1 Data (XLSX)

S2 Data (XLSX)

S3 Data (XLS)

S1 Questionnaire (DOCX)
