# Supplementary material for: Acute gastroenteritis and the usage pattern of antibiotics and traditional herbal medications for its management in a Nigerian community
Source: PLoS One. 2021 Oct 4;16(10):e0257837. doi: 10.1371/journal.pone.0257837 (PMC8490005; doi:10.1371/journal.pone.0257837)
Supplement: S1 Table — (DOCX) [file pone.0257837.s001.docx]

**S1 Table**

| **SN** | **Plant** | | | | **Harvest Details** | | | | **Ascension number** |
| --- | --- | --- | --- | --- | --- | --- | --- | --- | --- |
|  | **Local Name** | **Scientific Name** | **Part** | **Latitude** | | **Longitude** | **Date** |  | |
|  | Akogun | *Aristolochia ringens* | Root | 9.09557 | | 4.82406 | 05/12/20 | UILH/011/1005/2020 | |
|  | Dongoyaro (Neem tree) | *Azadirachta indica* | Leaves | 8.48025 | | 4.67772 | 19/10/20 | UILH/004/986/2020 | |
|  | Akintola | *Chromolaena odorata* | Leaves | 8.48019 | | 4.67819 | 22/10/20 | UILH/001/1281/2020 | |
|  | Igbaluwere | *Etanda africana* | Bark | 8.46338 | | 4.63694 | 04/12/20 | UILH/013/1668/2020 | |
|  | Igi odan | *Ficus capensis* | Leaves | 8.48048 | | 4.673389 | 01/12/20 | UILH/007/1417/2020 | |
|  | Igi odan | *Ficus vogelii* (syn. *F. lutea*) | Leaves | 8.50780 | | 4.55508 | 22/10/20  27/10/20  16/11/20 |  | |
|  | Mango | *Mangifera indica* | Leaves | 8.48101 | | 4.67713 | 19/10/20 | UILH/003/1080/2020 | |
|  | Ejinrinle | *Momordica charantia* | Leaves | 8.50780 | | 4.55508 | 28/10/20  30/11/20 | UILH/008/1292/2020 | |
|  | Efirin | *Ocimum gratisimum* | Leaves | 8.47990 | | 4.67825 | 23/10/20 | UILH/005/984/2020 | |
|  | Ajarere | *Senna alata* | Leaves | 8.47182 | | 4.65544 | 01/12/20 | UILH/009/1069/2020 | |
|  | Sorghum | *Sorghum bicolor* | Sheath | 8.46846 | | 4.64921 | 01/12/20 | UILH/010/1172/2020 | |
|  | Ewuro (Bitter leaf) | *Vernonia amygdalina* | Leaves | 8.48468 | | 4.58397 | 18/10/20  23/10/20 | UILH/002/972/2020 | |
