# Supplementary figures and images for: Acute gastroenteritis and the usage pattern of antibiotics and traditional herbal medications for its management in a Nigerian community
Source: PLoS One. 2021 Oct 4;16(10):e0257837. doi: 10.1371/journal.pone.0257837 (PMC8490005; doi:10.1371/journal.pone.0257837)

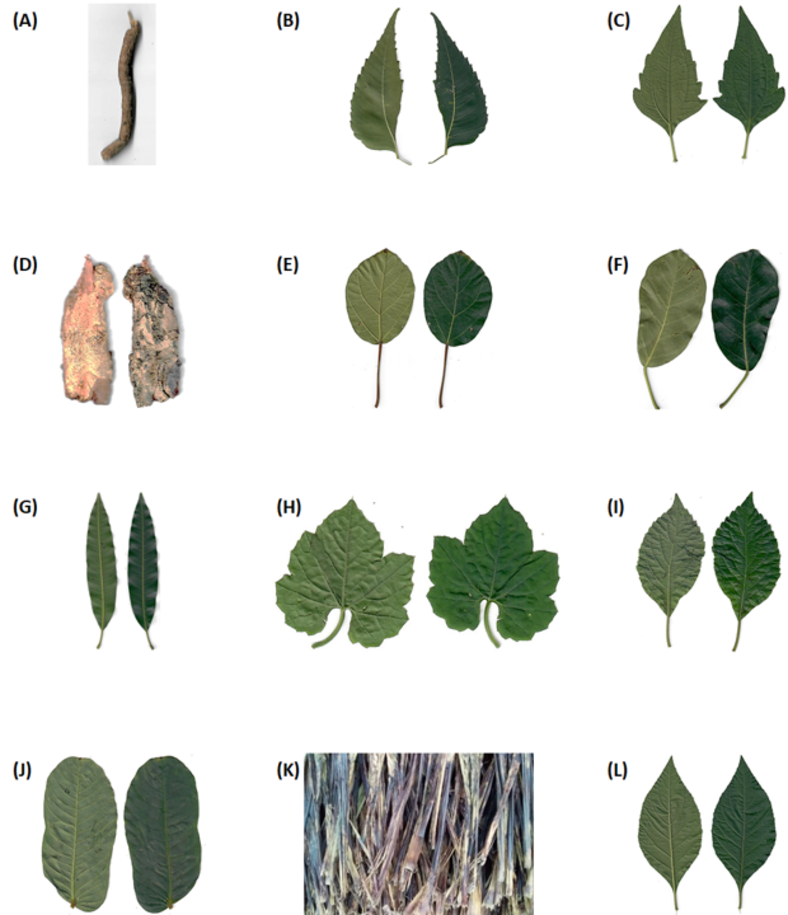

Supplement: S1 Fig — Figure shows (A) Image of Aristolochia ringens root (B) Upper and lower images of Azadirachta indica leaf (C) Upper and lower images of Chromolaena odorata leaf (D) Upper and lower images of Etanda africana leaf (E) Upper and lower of Ficus capensis leaf (F) Upper and lower images of Ficus vogelii (syn. F. lutea) leaf (G) Upper and lower images of Mangifera indica leaf (H) Upper and lower images of Momordica charantia leaf (I) Upper and lower images of Ocimum gratisimum leaf (J) Upper and lower images of Senna alata (K) Images of Sorghum bicolor sheath (L) Upper and lower images of Vernonia amygdalina leaf. (TIF) [file pone.0257837.s002.tif]
