# Supplementary material for: Acute gastroenteritis and the usage pattern of antibiotics and traditional herbal medications for its management in a Nigerian community
Source: PLoS One. 2021 Oct 4;16(10):e0257837. doi: 10.1371/journal.pone.0257837 (PMC8490005; doi:10.1371/journal.pone.0257837)
Supplement: S1 Questionnaire — (DOCX) [file pone.0257837.s006.docx]

**UNIVERSITY OF ILORIN**

**COLLEGE OF HEALTH SCIENCES**

**FACULTY OF BASIC MEDICAL SCIENCES**

**ATTITUDE TOWARDS ACUTE VIRAL GASTROENTERITIS IN KWARA STATE**

**INFORMED CONSENT FORM**

**Invitation to participate**

You are invited to participate in a research study to investigate the practice of patients with acute gastroenteritis. This ward has been selected among others in Kwara, State, Nigeria. This study does not involve any medical treatments or procedures, neither does it involve any kind of intervention or associated risk. You will be asked some questions about acute gastroenteritis by a member of the research team who will also describe the study, its purpose and potential benefits to you. Once you understand the study, you will be asked if you wish to participate. If you do, you will be asked to sign this consent form to indicate your consent.

**Benefits**

There are no direct and immediate benefits from participation in this study; however, knowledge we gained will help us develop more effective health intervention.

**Confidentiality**

Information obtained will be kept with uttermost confidentiality. No individual identifying information will be kept about you such that anyone would be able to connect your name to the proceedings of this study.

**Questions**

Before you agree to participate in this study, we want to give you the opportunity to ask questions on any aspects of this study that are not clear to you. You may take as much time as necessary to think this over. Do you have any questions at this point?

**Voluntary participation**

You may decide to participate in this study or not with no consequences. You are free to withdraw from the study at any time with no consequences.

**Rights**

You understand that you are free to ask questions before signing this form to participate in the study. Do you have any questions about your participation?

Please indicate (by ticking) your preference for one or two of the choices below.

( ) I do not accept to participate in this study.

( ) I accept to participate in this study.

**Authorization**

I, the undersigned, have read and understand the purpose, risks, and benefits of this study and am willing to participate in it.

| **----------------------------------------------** |  | **--------------------------------------------** |
| --- | --- | --- |
| **Participant/ Guardian’s Signature & Date** |  | **Research Assistant’s Signature & Date** |

**QUESTIONNAIRE**

**ACUTE VIRAL GASTROENTERITIS IN KWARA STATE**

**S/N ………………………**

| **Age** | **Religion (please tick)** | | | | | **Sex (please tick)** | | | |
| --- | --- | --- | --- | --- | --- | --- | --- | --- | --- |
|  | Christian ( ) | Traditional ( ) | | Muslim ( ) | | Male ( ) | | Female ( ) | |
| **Residential area/ LGA** |  | | | | | | | | |
|  | | | | | | | | | |
| 1. **Dysentery** | | | Yes ( ) | | No ( ) | | Duration | |  |
| 1. **Diarrhoea** | | | Yes ( ) | | No ( ) | | Duration | |  |
| 1. **Average number of stooling per day** | | |  | | | | | | |
| 1. **Vomiting?** | | | Yes ( ) | | No ( ) | | Duration | |  |
| 1. **Fever** | | | Yes ( ) | | No ( ) | | Duration | |  |
| 1. **Rashes** | | | Yes ( ) | | No ( ) | | Duration | |  |
| 1. **Aching limbs** | | | Yes ( ) | | No ( ) | | Duration | |  |
| 1. **Stomach cramps** | | | Yes ( ) | | No ( ) | | Duration | |  |
| 1. **Is the patient on traditional or local herbal medications?** | | | Yes ( ) | | No ( ) | | Duration | |  |
| 1. **If ‘Yes’ (in 9 above), what is the name of the herb(s) if known?** | | | (A) | | | | | | |
|  |  |  | (B) | | | | | | |
|  |  |  | (C) | | | | | | |
| 1. **If you answered ‘Yes’ to question No. 9 above, what is the source of prescription for the herbal medication? (please tick)** | | | **Herbalist** | | **Health Worker** | | **Other** | | **Self** |
|  |  |  |  | |  | |  | |  |
| 1. **Is the patient on antibiotics?** | | | Yes ( ) | | No ( ) | | Duration | |  |
| 1. **If you answered ‘Yes’ to question No. 12 above, what is the name of the antibiotic(s) if known?** | | | (A) | | | | | | |
|  |  |  | (B) | | | | | | |
|  |  |  | (C) | | | | | | |
| 1. **If you answered ‘Yes’ to question No. 12 above, what is the source of the prescription for antibiotics? (please tick)** | | | **Doctor** | | **Pharmacist** | | **Health Worker** | | **Self** |
|  |  |  |  | |  | |  | |  |
